# Elevated Contact Stresses Compromise Activity-Mediated Cartilage Rehydration but not Lubrication

**DOI:** 10.1007/s10439-025-03708-z

**Published:** 2025-05-03

**Authors:** Shamimur R. Akanda, Meghan E. Kupratis, Arnab Bhattacharjee, Jamie Benson, David L. Burris, Christopher Price

**Affiliations:** 1 Mechanical Engineering, University of Delaware, Newark, DE USA; 2 Biomedical Engineering Department, University of Delaware, 590 Avenue 1743, Newark, DE USA

**Keywords:** Cartilage tribology, Interstitial lubrication, Cartilage mechanical properties, Cartilage indentation, Mismatch disease

## Abstract

**Purpose:**

Understanding how obesity—a key risk factor for osteoarthritis—effects articular cartilage function is critical to understand OA pathoetiology. Cartilage, a biphasic material, supports vanishingly low friction coefficients in vivo, but is tribomechanically compromised by load-induced interstitial pressure/lubrication loss. To maintain tribomechanical function, cartilage must recover fluid lost to habitual/average contact stresses, a problem obesity likely exacerbates. Recently, we have shown that articulation/sliding drives robust interstitial fluid recovery and indefinite maintenance of biofidelic tissue strains and frictions through generation of hydrodynamic pressures within cartilage contact interfaces, i.e., via ‘tribological rehydration.’ However, the impact of elevated contact stresses on tribological rehydration and cartilage’s function/lubrication remains unknown.

**Methods:**

Using our convergent stationary contact area (cSCA) testing approach on ovine stifle cartilage explants bathed in PBS, we aimed to elucidate several points: (1) the effect of elevated contact stress on tribological rehydration during high-speed articulation, and how (2) cartilage material properties and (3) sliding speed influence contact stress-dependent fluid exudation, rehydration, and lubrication.

**Results:**

Overall, we identified that (i) contact stress, across a narrow range, and (ii) static loading time are key controllers of tribological rehydration magnitude, compression accumulation, and equilibrium/total compression under biofidelic cSCA loading and sliding conditions. However, over the range tested (i.e., 0.2–0.8 MPa), (iii) contact stresses had no appreciable effect on cartilage’s remarkable lubricity in the cSCA.

**Conclusions:**

These results show that obesity is likely to directly physically impair articular cartilage function, and that obesity-driven tissue compression/strain, and not friction *per se*, may be the primary mechanical driver of cartilage dysfunction and OA risk.

**Supplementary Information:**

The online version contains supplementary material available at 10.1007/s10439-025-03708-z.

## Introduction

Evidence points to osteoarthritis (OA) prevalence having doubled—since the 1950s in Western society—relative to pre-industrial/ancient societies [1], which has been postulated to result from our ‘bodies being inadequately adapted to modern conditions not encountered in our evolutionary past’ [2]. Thus, OA may be viewed, like many modern diseases (e.g., cardiovascular disease, obesity, diabetes, etc.), as an evolutionary “mismatch” disease [1]. To better understand OA etiology, and reduce its prevalence, the modern mismatch(es) our joints are inadequately adapted to need to be defined.

Two factors thought to drive increasing OA prevalence are obesity and physical inactivity [2–5], which have increased dramatically in postindustrial society [2, 6, 7]. Elevated body mass, obesogenic diets, chronic inflammation, and sedentariness are well-accepted OA risk factors [2, 8], with the effects of systemic obesity-associated molecular mechanisms (e.g., adipokines, metabolic stress/syndrome, metaflammation, etc.) on cartilage physiology being the focus of extensive study [9, 10]. Nevertheless, it remains unclear precisely how obesogenic comorbidities influence joint health. For example, Berenbaum suggests that when BMI/obesity is controlled for, physical inactivity may represent a separate OA risk factor [2]. Given these findings, and the intuition that obesity and inactivity generate abnormal joint loading environments, it is surprising that investigations into the purely physical effects of obesity and inactivity on cartilage function have seen scant focus. While studies demonstrate that individuals with elevated BMI experience larger cartilage strains and joint contact stresses [11, 12], how such “mismatched” loading conditions mechanistically influence in vivo cartilage function—especially its tribomechanics—has only been speculated upon to date.

It is universally accepted that healthy articular cartilage has phenomenal load bearing and lubricating abilities [13, 14]. These capabilities have been attributed to several tribomechanical and lubrication mechanisms (e.g., weeping, boundary lubrication, fluid film, interstitial lubrication, etc.) [13, 15–20], but appear to be primarily underpinned by cartilage’s multi-phasic composition [19]. When initially loaded, cartilage generates transient high interstitial fluid pressures that limit tissue compression/strain and shear/friction (friction coefficients [*μ*] ~ 0.02 can be sustained through interstitial lubrication) [19]. Thus, on one hand, healthy cartilage appears functionally optimized to support transiently applied peak contact stresses that are an order of magnitude larger (10–20 MPa) [21] than the tissues’ equilibrium compressive modulus (0.4–1 MPa)[22–24]. On the other hand, quasi-static loading drives time-dependent fluid exudations, tissue compression, and loss of fluid load support (FLS) and interstitial lubrication, all detrimental to cartilage function, lubricity, and health [19]. In vivo, cartilage is habitually exposed to non-zero quasi-static average contact pressures predicted to range from 0.1 to 2 MPa [21]. At these contact stresses, time-dependent defeat of interstitial lubrication by exudative flows generate unremarkable frictions (*μ*  ~ 0.4) [25, 26].

Due to its construction, cartilage should be quite sensitive to elevated habitual contact stresses associated with obesity [27], an observation that cannot be overlooked in the study of cartilage dysfunction. The accumulation of time-dependent cartilage compressions and loss of FLS due to habitual non-zero contact stresses must be counteracted by interstitial fluid recovery to preserve cartilage’s fluid pressure-dependent functions. Fortunately, studies on intact joints and cartilage explants demonstrate articulation to be a strong modulator of fluid recovery [28]. Such fluid recovery was once thought to occur solely through ‘passive’ mechanisms, during cartilage unloading and/or ‘bath exposure’ during joint articulation [29–32]. However, we have recently demonstrated a complementary mechanism through which articulation promotes active interstitial fluid recovery. On re-examining the convergent stationary contact area (cSCA) testing configuration (comprising a large convex cartilage-on-flat glass contact) [17, 25] under biofidelic sliding speeds (> 30 mm/s) and contact pressures (~ 0.25 MPa), we have observed that sliding promotes robust recovery of interstitial fluid, FLS, and interstitial lubrication [24–26, 33–37]. Contact uncovering and contact migration do not occur in the cSCA and can’t contribute to fluid recovery. Instead, interfacial sliding generates hydrodynamic pressures in the convergent contact that drives fluid recovery and rescues/prevents load-induced tissue compression and compromise of interstitial lubrication, a phenomenon we have termed ‘tribological rehydration’ [25]. Because of ‘tribological rehydration,’ the saline-bathed cSCA is unique in its ability to sustain—near indefinitely—biofidelic tissue strains (~ 5 to 15%), frictions (~ 0.03), and FLS (< 95%) on the benchtop [25], unlike a traditional stationary contact area (SCA) configuration [25, 26, 35, 38].

Tribological rehydration thus represents a novel phenomenon by which joint articulation/activity can rescue and sustain articular cartilage function, one underpinned by competition between contact pressure-driven fluid exudation and sliding-mediated fluid recovery [25, 26]. Thus, it can be intuited that elevated habitual contact stresses—such those associated with obesity [39]—would represent a negative, but modifiable, influence on activity-driven interstitial fluid recovery and cartilage function. The cSCA, by facilitating predictable sliding-mediated interstitial fluid and lubrication recovery, represents the ideal tool to investigate the influence of contact stress and activity level “mismatches” on articular cartilage function. The goal of this study was to investigate the influence of elevated contact stresses—a surrogate for body mass/obesity—on sliding-induced tribological rehydration under lubrication by phosphate buffered saline (PBS). Specifically, we sought to (1) investigate the effect of contact stress on the competitive balance between fluid exudation and rehydration during cSCA sliding), and establish how (2) intrinsic cartilage properties (i.e., moduli, permeability, etc.) and (3) sliding speed—a rough surrogate of activity level—influence cartilage fluid exudation, rehydration, and lubrication behaviors. To accomplish these goals, we leveraged cSCA explants harvested from ovine (sheep) femoral condyles to study the effects of elevated contact stresses on sliding-induced tribological rehydration and lubricity, while bathed in PBS.

## Methods

### Tissue Specimens

Stifle joints from normal healthy lambs (~10-12 months of age and of mixed sex, *n* = 12) were obtained—frozen—from a local abattoir (Bowman’s Butcher Shop, MD). Osteochondral cores having the geometry necessary for the cSCA configuration (∅12 mm, ~15 mm tall) were extracted from the centerline of the femoral condyles using a coring bit and drill (*n* = 29 cores/explants) [36]. Upon removal, the direction of in vivo articulation was inscribed on the base of the bone core for reference, and the specimens were stored in 1X PBS (270 mOsm) with protease inhibitors (hereon referred to as PBS) [40] at 4 °C for less than 48 hr before testing. Explants were randomly assigned to one of three studies (see below).

### Tribological Tests

**Fig. 1:**
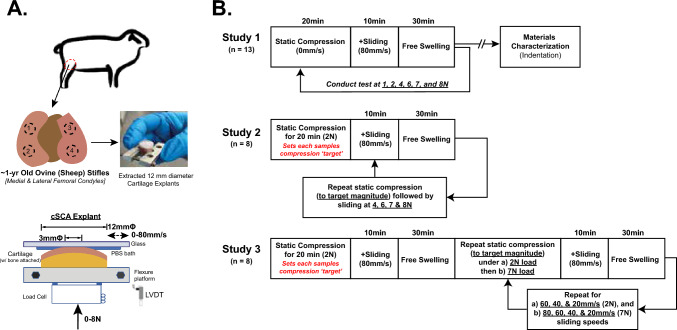
Experimental design and sample preparation. **A** Schematic illustrating the location, number, and geometry of cSCA explants extracted from the femoral condyles of ~1-year old ovine stifle joints. Explant diameters (∅12 mm) were chosen such that the convex curvature necessary to generate convergent wedges at the contact periphery was preserved, resulting in an ~ 3 mm∅ contact interface for these cSCA explants when loaded (based on a circular approximation of the measured *in situ* contact area). **B** Three different characterization protocols (Studies 1, 2, & 3) assessed sliding-induced tribological rehydration of cSCA explants under varying applied loads (0–8 N), compression states (variable FLS levels), and sliding speeds (0 mm/s, static loading; 80 mm/s, Studies 1 and 2; and 20–80 mm/s, Study 3), respectively.

A linearly reciprocating tribometer was used to characterize cSCA cartilage tribology in the principle direction of articulation [25]. In this configuration, the curved-on-flat stationary contact between the cartilage explant and glass counterface forms convergent wedges at the contact periphery because the diameter of the cartilage-on-glass contact region (ca. ∅3 mm) [36] is less than explant diameter (∅12 mm). Pressurization of the bathing fluid within these ‘wedges’ hydrodynamically facilitates tribological rehydration during sliding [25]. Cartilage *compression* (*δ*), *normal force* (*F*_N_), *friction force* (*F*_F_), and *kinetic frictional coefficients* (*µ*_*k *_= $\frac{{F}_{F}}{{F}_{N}})$ were continuously sampled at 500 Hz. Data were automatically extracted over the central 25% of each 20-mm-long sliding track, with the positive and negative sliding directions averaged to obtain a single-cycle datum. Three different studies (Figure 1), applying variable load magnitudes, static loading times, and sliding speeds, were utilized to assess how contact stresses affect tribological rehydration. Samples were hydrated/bathed in PBS for the duration of testing and allowed to free swell in PBS for > 30 min between tests.

### Study 1: Effect of Contact Stress on Tribological Rehydration Under Quasi-Static Equilibrium Compression

The first study (Figure 1B) characterized the influence of contact load (and thus contact stress) on tribological rehydration behaviors when high-speed sliding was initiated from quasi-static equilibrium compression. For ovine explants, the characteristic unconfined compression stress relaxation time is ~ 15 minutes (*τ* = *a*^2^/*H*_a_*k*; for *a* = 1.5 mm, *H*_a_ = 0.4 MPa, and *k* = 6 × 10^−15^ m^4^/Ns) [24, 36, 41]; as such, each explant (n = 13) was compressed for 20 minutes at each of six different *F*_N_ values (1, 2, 4, 6, 7, 8 N, corresponding to ~ 0.16, 0.26, 0.38, 0.46, 0.49, and 0.53 MPa; *see contact area/stress measurement below*), followed by 10 minutes of sliding at 80 mm/s under continuous compression. 20 minutes of static loading was implemented to permit a reasonable approximation of quasi-static tissue compression—based on the above *τ* [41], while a sliding speed of 80 mm/s was used to approximate the average speed of articulation of the femoral condyle past the tibial plateau during (walking) gait in humans [34].

### Study 2: Effect of Increased Contact Stress on Tribological Rehydration from a Fixed Initial Compression

Next, we examined the effect of contact stress on sliding-induced tribological rehydration under varied initial FLS. A separate set of explants (*n* = 8) were compressed at 2 N for 20 min to define a sample-specific target compression value (~ 60 to 100 µm, corresponding to approximately 20 to 40% strain), then slid at 80 mm/s for 10 minutes. Tribological characterization was then repeated, sequentially, following free-swelling, under applied loads of 4, 6, 7, and 8 N, with sliding being initiated upon attaining the 2 N target compression. Because compression time decreased with increasing load, FLS at the start-of-sliding varied (from low to high, respectively), while initial compression magnitude remained fixed.

### Study 3: Effect of Contact Stress and Sliding Speed on Tribological Rehydration:

Finally, we investigated whether the effects of contact stress on tribological rehydration vary with sliding speed in a third set of explants (*n* = 8). Target compression values at 2 and 7N were established as in Study 2, followed by tribological characterization at 80 mm/s for 10 minutes. Samples were free swelled for 30 minutes before repeating the characterization at different sliding speeds (60, 40, and 20 mm/s). A similar tribological characterization was performed under 7N load (Figure 1B).

### In Situ Contact Area Measurement

Following tribological testing, *load* (*F*_N_)*-dependent contact areas* (*A*) were determined by introducing India ink into the bath solution [26, 42] to observe and digitally image the contact area at increasing loads; the India ink is excluded from the cartilage-on-glass contact. From these images the contact was traced, and *load-dependent* *A *and *contact stress* (*σ* = *F*_*N*_/*A*) were calculated via MATLAB (2021a, The MathWorks).

### Hertzian Indentation

A custom microtribometer was used to quantify biphasic tissue properties of explants from Study 1. Details pertaining to this device are described in the Supplemental Methods. Briefly, the indenter consisted of an encoded vertical nanopositioning stage, a cantilever beam-based load cell (spring constant = 1165N/m) with an attached spherical glass probe (∅ = 1.5 mm), and capacitive displacement sensors to measure beam deflection. Cartilage specimens were indented at a rate of 50 µm/s until a target force of ~ 20mN was generated and then allowed to undergo stress relaxation. The resultant force–displacement curves were fit to Hertz’s equation (${E}_{C}= \frac{3}{4}{F}_{n}{R}^{-.5}{\delta }^{-1.5}$) and Hertz Biphasic theory was used to calculate *compressive modulus* (*Ε*_y*−*_), *tensile modulus* (*Ε*_y*+*_), and the *unstrained permeability* (*k*_0_) [43].

### Cartilage Thickness Measurement

After testing, explants were bisected through the center of the cSCA contact and imaged using a digital stereomicroscope to visualize the articular cartilage geometry. A custom MATLAB code [34] was used to trace the cartilage and calculate average *cartilage thickness* (*h*) via Euclidean distance transform.

### Data Analysis

**Fig. 2:**
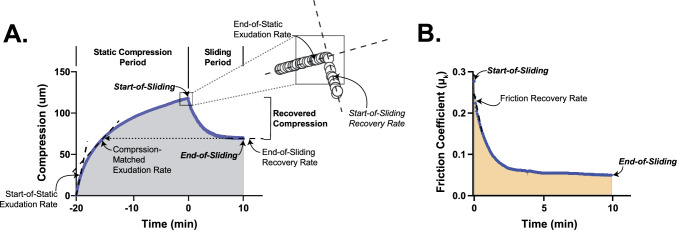
Data extracted from cSCA-based tribology characterization tests. Representative **A** compression and **B** kinetic coefficient of friction traces illustrating the response of a cSCA explant to static loading and sliding-mediated tribological rehydration. Shown is an explant subjected to 20 min of static loading (2 N) followed by 10 minutes of high-speed (80 mm/s) sliding (in PBS) superimposed upon the 2 N load. Tribomechanical parameters extracted from these tests included compression (*ε*), magnitudes and friction coefficients (*μ*_k_), during the first and last reciprocating sliding cycles (start- and end-of-sliding values, respectively); recovered compressions (i.e., start-of-sliding minus end-of sliding compression); and rates of fluid exudation, compression recovery, and rehydration at the start- and end-of-static loading, and the start- and end-of-sliding. Rehydration rates during sliding were defined, due to conservation of volume, as the compression-matched exudation rate minus the sliding-mediated recovery rate.

Tribomechanical measures, including *compression* (*δ*) and *kinetic friction coefficient* (*μ*_k_), were extracted from the raw data as described previously [34]. Datum corresponding to *start-of-sliding* (first reciprocal sliding cycle) and *end-of-sliding* (last reciprocal sliding cycle) values are shown in Figure 2. Sliding-induced *compression recovery* was defined as the difference between the start- and end-of-sliding compressions. *Percent compression recovery* as = $\frac{Compression Recovery}{Total Compression}\times 100\%$. Compressive *strain* was defined by normalizing compression by cartilage thickness (*ε* = δ/*h*).

Rates of load-induced fluid exudation and sliding-induced tribological rehydration were calculated using linear fits of time-dependent tissue compression [32]. *Start-* and *end-of-static* *exudation* and *start-* and *end-of-sliding recovery rates* were defined as the slope of the linear compression fit at the start- and end-of-static loading and start- and end-of-sliding, respectively (each fit over 30 s). Rehydration rates were calculated as the difference between load-induced exudation and sliding-induced recovery rates; during sliding, exudation rates were defined by referencing compression-matched exudation rates during the preceding static loading period [32].

### Statistical Analysis

Tribological outcomes were fit to mixed effects (ME) models as functions of load, contact stress, and sliding speed [R package Ime4 (version 1.1.21)]. This approach facilitated comparison of speed- and load-dependent characteristics of tribological rehydration among samples by accounting for variation arising from both fixed effects (e.g., load/contact stress) and random effects (e.g., sample identity) [36, 44]. Tribomechanical outcomes were then fit to linear (*y* = *ax* + *b*) or 2nd-order (*y* = *ax* + *bx*^2^) ME models, as established by Akaike information criterion (AIC) analysis for distinguishing the likelihood of a specific model describing tribomechanical outcomes as functions of load or stress (see Supplemental Methods) [45]. Relationships (i) among tribomechanical outcomes and tribomechanical inputs (i.e., applied load, contact stress, sliding speed, etc.) were evaluated using linear regression and mixed-effect [ME] modeling approaches (GraphPad Prism ver. 10.2.2) and (ii) among tribomechanical outcomes and cartilage tissue properties via Pearson correlation analysis [R package corrplot (ver. 0.84)].

## Results

### Study 1: Effect of Contact Stress on Tribological Rehydration When Slid from a Quasi-Static Compression State

**Fig. 3:**
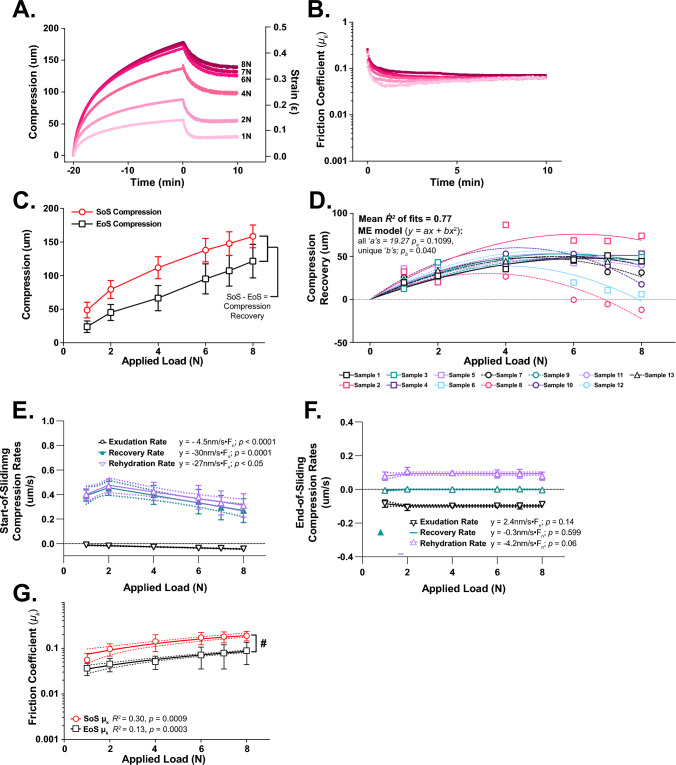
Load-dependent compression, tribological rehydration, and friction behaviors of cSCA explants slid at 80 mm/s starting from quasi-static compression (Study 1, *n* = 13 explants).** A** Compression/strain and **B** friction responses from a representative ovine cSCA explant subjected to static compression for 20 minutes followed by 10 minutes of 80 mm/s sliding at six different loads (1–8 N). Samples were unloaded and allowed to free swell in PBS for 30 minutes between loads. **C** Start- and end-of-sliding compressions (SoS & EoS, respectively) increased with applied load. Average end-of-sliding compressions were lower than start-of-sliding compression due to tribological rehydration-mediated compression recovery. Data are shown as mean ± 95% CI. **D** When plotted individually against applied load, specimen-specific load-dependent tribological rehydration behaviors (i.e., compression recoveries) were well described by a constrained 2nd-order polynomial (*y = ax + bx*^2^) ME. Positive compression recovery indicates net fluid recovery during sliding, negative recovery indicates net fluid exudation. Rates of compression recovery, and fluid exudation and rehydration in cSCA explants at the **E** start-of-sliding (initiated from quasi-static compression) and **F** end-of-sliding calculated for different applied loads. Solid lines indicate the best fit of a segmental linear regression; the dashed lines represent the 95% CI of the best fit. *N.b.*: the slopes of the best fit lines are indicated for load ≥ 2 N and reported as absolute values. **G** In our PBS-bathed contacts, start- and end-of-sliding friction increased modestly as applied load increased (*p*_slope_ = 0.009 and 0.003, respectively). Data points are shown as mean ± 95% CI; solid lines indicate the best fit linear regression of the data, and the dashed lines the 95% CI of this fit. *N.b.*: Data in G are shown on a log-normal plot; # - end-of-sliding friction coefficients were different from those at the start-of-sliding, *p* = 0.0012, RMME

The influence of applied load—and thus contact stress—on a representative cSCA explant’s compression, compression recovery, and lubrication response when slid—at 80 mm/s in PBS—from quasi-static compression is shown in Fig. 3A, B. Higher loads generated greater rates of fluid exudation upon loading (i.e., *start-of-static*, SFig. 2A) and larger cartilage compressions after 20 minutes (i.e., *start-of-sliding*, Fig. 3C, n = 13 explants; see SFig. 2B for individual explant data), as expected. Nonetheless, at the end of the static loading period (i.e., *start-of-sliding*) exudation rates were universally < 1/15th of their *start-of-static* rates (Fig. 3E); supporting the notion that sliding was initiated from a condition of quasi-static compression and low *FLS* levels. Subsequently, following 10 minutes of 80 mm/s sliding, tribological rehydration was apparent, on average, across all loads (Fig. 3C & SFig. 2C). However, mixed effect [ME] modeling of compression recovery behaviors vs. applied load revealed several key load-dependent characteristics of sliding-induced tribological rehydration (Fig. 3D). First, *compression recovery* was small (but always positive) at loads ≤ 2 N and maximal at intermediate loads (4-6N). Second, at loads > 6N, compression recovery diminished and was occasionally negative.

The influence of applied load on competition amid sliding-mediated tissue rehydration and load-induced fluid loss was evident among rates of tissue *exudation*, compression *recovery*, and tribological *rehydration* at the start- and end-of-80 mm/s sliding (Figure 3E–F). *Start-of-sliding exudation rates* increased modestly (becoming more negative) with load. *Start-of-sliding recovery rates* increased from 1 to 2 N and then decreased from 2 to 8 N. *Rehydration rates,* reflecting the difference between *exudation* and *recovery rates*, tracked with, but were higher than recovery rates, due to volume conservation. At the end-of-sliding, all samples achieved “dynamic sliding equilibria” compressions, reflecting *recovery rates* of zero due to exudation and rehydration competitively “offsetting” each other.

When rapidly slid from quasi-static compression and in PBS, cSCA explant lubricity and lubrication recovery were influenced by applied load magnitude (Fig. 3G). With increasing loads, FLS loss drove increased *start-of-sliding friction coefficients*, while sliding-induced tribological rehydration drove significant recoveries of cartilage lubricity across all loads. As a result, *end-of-sliding frictions* were always significantly lower than their respective 80 mm/s *start-of-sliding* values. Whereas both *start-* and *end-of-sliding frictions* increased modestly with load, the effect of unit increases in applied load on *start-of-sliding friction* was more than twice that on *end-of-sliding friction* (*y* = 0.018*L + 0.053 vs. *y* = 0.0072*L + 0.027, respectively).

**Fig. 4:**
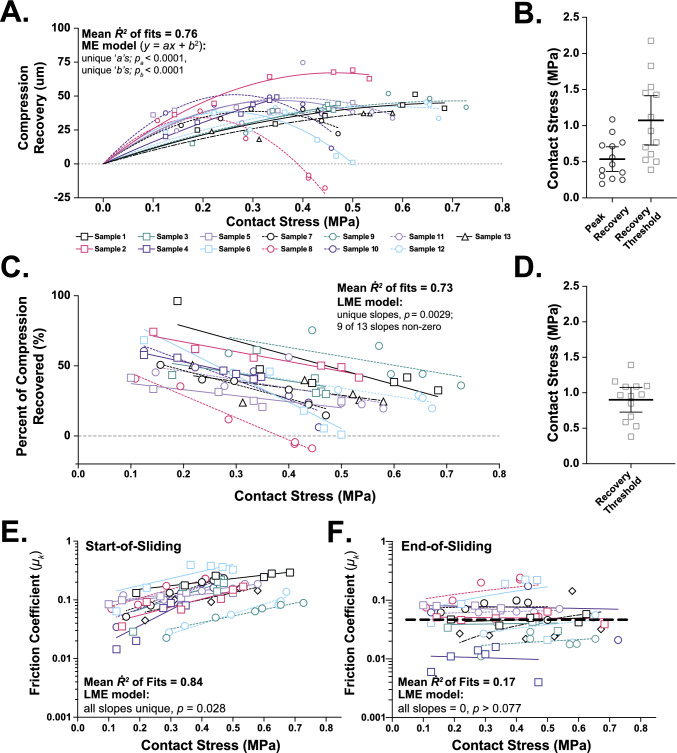
Contact stress-dependent compression recovery and frictional behaviors of cSCA explants slid at 80 mm/s from quasi-static compression. **A** Sliding-mediated compression recovery (at 80 mm/s) plotted against contact stress for the *n* = 13 ovine stifle cSCA explants examined in Study 1. Individual explants are denoted with different symbols (as in Figure 3D) and fit with constrained 2nd-order polynomials (*y = ax + bx*^2^) ME to highlight explant-specific tribological rehydration behaviors. From each individual compression recovery vs. contact stress curve **B** the contact stresses at which peak compression recoveries and zero net compression recovery (i.e., the recovery threshold) occurred could be extrapolated, ~ 0.5 ± 0.28 and ~ 1.0 ± 0.56 MPa, respectively. **C** Plotting percent of static compression recovered by sliding against contact stress confirmed inverse, explant-specific, relationships between net tribological rehydration and contact stress (LME: *p*_slopes_ = 0.0029). **D** Compression recovery threshold stresses calculated based on linear fits of the percent compression recovery vs. contact stress curves (~0.9 ± 0.28 MPa) aligned well with those derived from compression recovery fits. Kinetic coefficient of frictions at the **E** start- and **F** end-of-sliding plotted across contact stresses. While start-of-sliding frictions tended to increase modestly with contact stress across all explants (LME: *p*_slope_ = 0.028), end-of-sliding frictions were largely insensitive to contact stress (LME: *p*_slope_ > 0.07, *R*^2^* = *0.17, slope = 0)

ME modeling of contact stress-dependent cSCA sliding outcomes revealed several effects of increasing contact stresses on cartilage compression (SFig. 3A, B), tribological rehydration, and lubrication behaviors (Fig. 4) when slid from quasi-static compression. Compression recovery vs. start-of-sliding contact stress was best fit by a constrained second-order polynomial (average *R*^2^ = 0.76; Fig. 4A). Estimation of each fit’s peak and root identified their mean *peak compression recovery* (~0.53 ± 0.17 MPa) and the *compression recovery threshold* (~ 1.1 ± 0.27 MPa) stresses, respectively (Fig. 4B), the latter being the stress at which compression behaviors transitioned from net recovery to net exudation under 80 mm/s sliding. A comparable estimate of *recovery threshold stress* was obtained by evaluating tribological rehydration in terms of *percent compression recovery* (*x*-intercept =  ~ 0.9 ± 0.18 MPa, Fig. 4C, D). When 80 mm/s sliding was initiated following 20 minutes of compression *start-of-sliding frictions* increased with contact stresses (Fig. 4E). In contrast, *end-of-sliding frictions* exhibited no relationship with stress across the range tested (Fig. 4F). Tribological rehydration and lubrication recovery behaviors presented as strains vs. load and stress can be found in SFig. 4.

### Biphasic Cartilage Material Properties Dictate Contact Stress-Dependent Tribomechanical Recovery

**Fig. 5:**
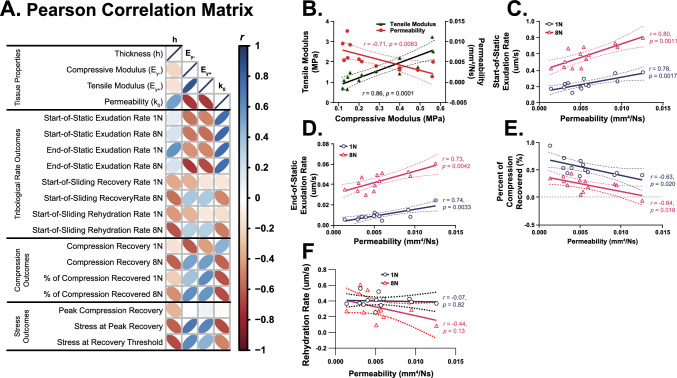
Relationships among cartilage biphasic tissue properties and cSCA explant tribomechanical outcomes under high-speed sliding initiated from quasi-static compression.** A** Pearson correlation matrix indicating linear relationships between fluid exudation, recovery, and rehydration rates; compression recoveries; and stress-dependent tribological rehydration characteristics and cartilage tissue properties from *n* = 13 explants examined in Study 1. For each pairwise comparison, both box color and oval ellipticity indicate Pearson correlation coefficient magnitude. **B–F** *X*–*Y* plots provide visualization of select pairwise linear correlations among tribomechanical outcomes and material properties. **B** Tensile moduli correlated positively, and unstrained permeability negatively with compressive modulus. **C** Start- and **D** end-of-static (*a.k.a.*, start-of-sliding) exudation rates correlated strongly with tissue permeability but differed by over an order of magnitude (note differences in y-axis scale). **E** Percent compression recovered by high-speed sliding was strongly and negatively associated with permeability. **F** Rehydration rates were not (i.e., non-significantly) correlated with tissue permeability. For each pairwise comparison, the Pearson correlation coefficient and corresponding *p*-value is provided along with the corresponding best fit line (solid) and 95% CI of this fit (dashed). For panels **C**–**F** only data for the experiments at 1 and 8 N are shown for the purpose of visual clarity. *N.b. Pearson correlation data for strain-based outcomes vs. tissue properties are provided in SFig 5*

**Fig. 6:**
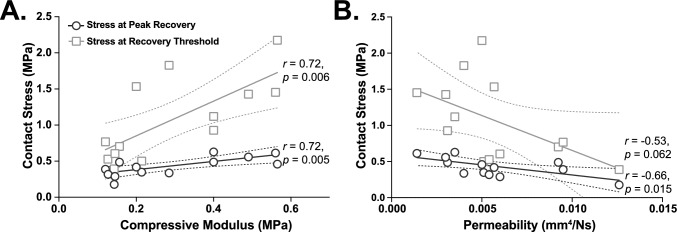
Correlation of contact stress-dependent tribological rehydration characteristics and biphasic tissue properties. The extrapolated contact stresses at which peak compression recovery and zero net compression recovery (i.e., the recovery threshold) of explants in Sudy 1 occurred were strongly and positively correlated with tissue compressive modulus (*r* = 0.72) (**A**) and inversely correlated with tissue permeability (*r* = − 0.53 to − 0.66) (**B**). For each comparison, the Pearson correlation coefficient and corresponding *p*-value are provided along with the corresponding best fit line (solid) and 95% CI of this fit (dashed)

Pearson correlational analysis among explant biphasic material properties (Supp Table 2) and tribomechanical behaviors (Study 1) highlighted several notable characteristics (Fig. 5A). Firstly, our explants exhibited the expected (i) tension-compression nonlinearity (*E*_*y+*_ [0.9–3.1 MPa] exceeded* E*_*y-*_ [0.1–0.6 MPa], by 5- to 6-fold), (ii) inverse correlation between permeability (*k*_0_) and *E*_*y−*_, and (iii) dependence of s*tart- and end-of-static exudation rates* on *k*_*0*_ (Fig. 5B–D). Regarding tribological rehydration, as *k*_0_ increased the percent *compression recovery* under 80 mm/s sliding decreased significantly (Fig. 5E); among 1 and 8 N loads these relationships had similar trajectories (i.e., slopes) and strengths (*r* ~  − 0.64), but different magnitudes. Weak-to-no correlation was observed between *rehydration rates* and *k*_0_ (Fig. 5F). Most revealing, strong covariances among stress-dependent tribological rehydration behavioral parameters (i.e., *peak compression recovery* and *compression recovery threshold stresses* at 80 mm/s) and *E*_*y-*_ and *k*_0_ were observed (Fig. 6). Performance of Spearman rank-order correlation revealed qualitatively similar monotonic relationship outcomes among our variables (not shown).

### Study 2: The Effect of Contact Stress on Tribological Rehydration Upon Sliding from Fixed Compression

**Fig. 7:**
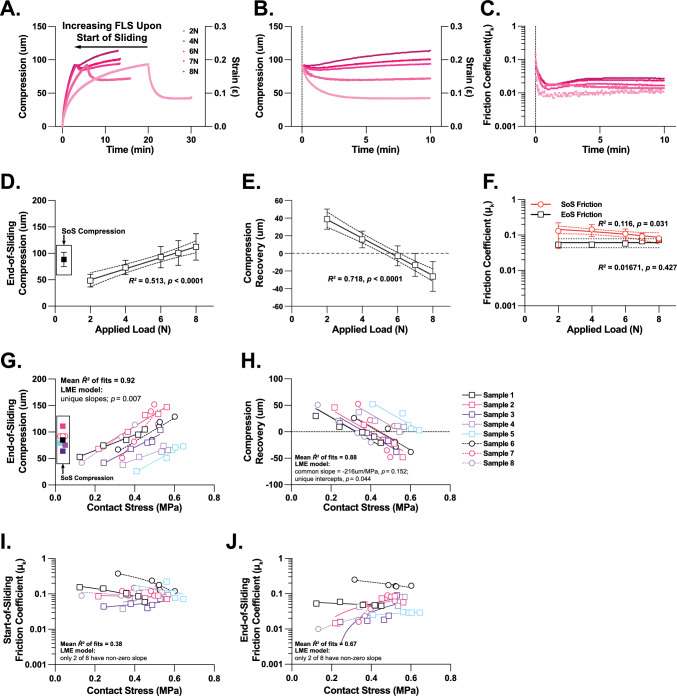
Load and contact stress-dependent cSCA explant tribomechanical behaviors when slid from intermediate levels of tissue compression/fluid load support (Study 2; n = 8 explants). Representative **A**, **B** compression and strain, and **C** friction responses of an ovine cartilage cSCA explant during tribological rehydration characterization under 80 mm/s sliding that was initiated from a fixed compression magnitude (i.e., variable FLS). Tribological testing comprised an initial static compression at 2 N for 20 minutes to establish the explants compression target followed by 10 minutes of sliding at 80 mm/s. Tests were repeated by compressing the explant to the target compression at 4, 6, 7, and 8 N followed by 10 minutes of sliding. Samples were unloaded and allowed to free swell in 1X PBS for 30 minutes between loads. **D** Across all explants, end-of-sliding compression increased linearly (*p*_slope_ < 0.0001) over the range of loads applied. The mean start-of-sliding compression targets, established by the initial 20 minutes of 2 N static compressions, are indicated in the inset for the *n* = 8 explants tested. **E** Recovered compression under 80 mm/s sliding decreased linearly across the applied loads (*p*_slope_ < 0.0001). **F** On average, start-of-sliding frictions tends to decrease as applied loads increased (and loading times decreased; *p*_slope_ = 0.031), while end-of-sliding frictions were largely invariant to load levels (*p*_*slope*_ = 0.427). Data points in D-F are shown as mean ± 95% CI, solid lines indicate the best fit linear regression; the dashed lines represent the 95% CI of the fit. When plotted individually, specimen-specific contact stress-dependent **G** end-of-sliding compression and **H** compression recovery behaviors were observed. LME analysis indicated that end-of-sliding compressions increased with contact stress (LME: *p*_slopes_ = 0.007), while compression recovery decreased. Individually, the slopes of the compression recovery vs. contact stress responses, but not the intercepts, were statistically indistinguishable (*p*_slopes_ = 0.152, slope = − 216 μm/MPa; *p*_intercepts_ = 0.044, respectively). Similarly, LME analysis indicated little to no effect of contact stress on both **I** start- and **J** end-of-sliding friction coefficients in PBS; only 2 of 8 explants exhibited slopes that were statistically different from zero (0)

As individually larger loads were applied to cartilage explants (*n* = 8), target compression levels (*ca.* ~ 84 ± 19 µm) were approached more rapidly (e.g., decreasing from ~ 20 to ~ 2.5 min for 2 vs. 8 N, respectively; Fig. 7A) because of elevated start-of-static exudation rates (SFig. 6A). This behavior allowed for interrogation of the effects of contact stress on tribological rehydration and PBS-mediated lubrication in the face of variable FLS levels (but similar compression magnitudes) (Fig. 7B, C). Under these conditions, *end-of-sliding compression* increased with applied load (Fig. 7D), while *compression recovery* decreased (Fig. 7E); negative *compression recovery*—or net exudation—occurred at loads ≥ 6N (see SFig 6C, D for individual explant data). This response was echoed in increasing suppression of sliding-mediated rehydration by load-driven exudation (SFig. 6B) during sliding. *Start-of-sliding* frictions decreased modestly with applied load (Fig. 7F), reflecting the influence of higher FLS levels accompanying ever shorter static loading periods on initial lubricity. In contrast, *end-of-sliding friction coefficients* were statistically indistinguishable across the range of applied loads (Fig. 7F).

ME modeling of Study 2 tribology outcomes with respect to start-of-sliding contact stresses highlighted clear stress-dependent impacts on *end-of-sliding compression* and *compression recovery*. *End-of-sliding compression* increased significantly with *contact stress* and in a manner unique to each specimen (Fig. 7G). In contrast, the effect of a unit increase in contact stress on *compression recovery* was indistinguishable among explants (i.e., a common slope was indicated; Fig. 7H). While the x-intercepts of the *compression recovery vs. contact stress* fits varied among specimens, an average threshold recovery stress of 0.45 ± 0.12 MPa could be projected. Consistent with load-dependent frictional outcomes, applied stresses had little effect on *start-* or *end-of-sliding friction coefficients* (Fig. 7I, J). cSCA sliding behaviors presented as strains vs. loads and stresses can be found in SFig. 6E–H.

### Study 3: The Influence of Contact Stress and Sliding Speed on Tribological Rehydration

**Fig. 8:**
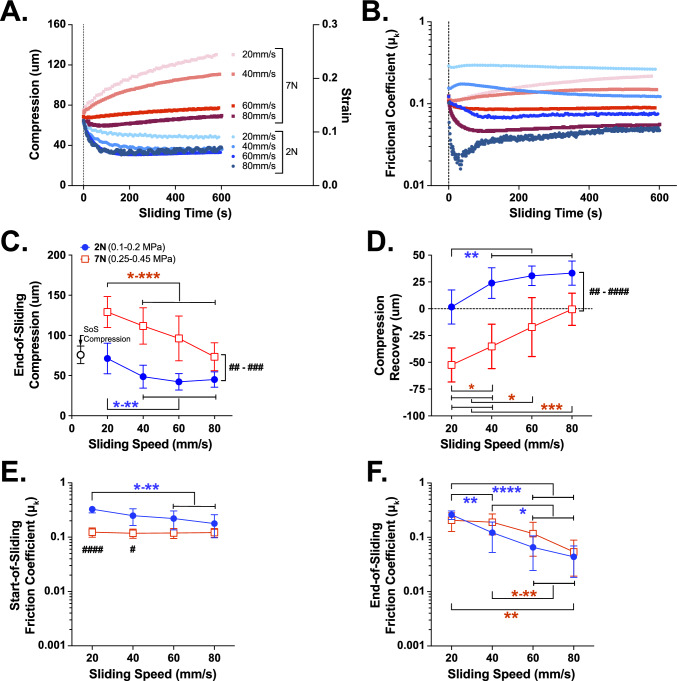
Load- and sliding speed-dependent cSCA explant tribology outcomes (study 3, *n* = 8 explants). Representative **A** compression/strain and **B** friction coefficient responses of an ovine cartilage explant characterized across a range of sliding speeds (80, 60, 40, 20 mm/s) under both 2 and 7 N loads. Each speed and load test was preceded by a period of static loading to allow initiation of sliding at the explant’s initial compression target value. **C** End-of-sliding compressions increased with increasing loads (*n* = 8, ^##^*p* < 0.01, ^###^*p* 0.001; RMME) and decreasing sliding speeds (*p < 0.05, ***p < 0.001). **D** Sliding-mediated compression recovery decreased with increasing loads (^##^*p* < 0.01, ^###^*p* 0.001; RMME) and slower sliding speeds (*p < 0.05, ***p < 0.001), exhibiting negative recoveries (i.e., net exudative behaviors) for sliding speeds < 80 mm/s at 7N. **E** Start-of-sliding frictions decreased modestly with sliding speeds under 2 N (*p < 0.05, ***p < 0.001), but not 7N loads . **F** End-of-sliding frictions increased significantly as sliding speeds decreased across all applied loads (*p < 0.05, ****p < 0.0001). Aside for start-of-sliding frictions under 20 and 40 mm/s sliding (^###^*p* < 0.0001, ^#^*p* < 0.01, respectively), neither start- nor end-of-sliding friction coefficients were markedly influenced by applied load at a given sliding speed. All data are shown as mean ± 95% CI. *N.b. Three-dimensional representations of the load- and sliding speed-dependent relationships obtained in Studies 2 & 3 can be visualized in SFig 8*

Lastly, we investigated the effects of sliding speed (80, 60, 40, vs. 20 mm/s) and load (2 vs. 7 N) on compression recovery and lubricity of PBS-bathed cSCA contacts (Fig. 8A, B). As in Study 2, sliding was initiated upon each explant achieving a target compression level (*ca.* 74.12 ± 12.3 µm); set by each initial 20 min of 2 N compression. Elevated loads and slower sliding speeds led to higher *end-of-sliding compressions,* and reduced *compression recoveries and recovery rates* (Fig. 8C, D). Under 2 N loads, 20 mm/s appeared to represent the average rehydration threshold speed (SFig 7A). In contrast, under 7N loads, negative *compression recoveries* were, on average, observed for sliding speeds < 80 mm/s (SFig 7B). *N.b. load- and sliding speed-dependent end-of-sliding strain and strain recovery responses are provided in SFig 7C, D. Start-of-sliding frictions* (i) at 2 N were higher than at 7N tests—because of depleted FLS effects—and (ii) were only sensitive to sliding speed at 2 N load (but only accounting for ~ 8% of the overall variance; Fig. 8E). *End-of-sliding friction coefficients* were iii) not influenced by applied load; iv) sliding speed was the major determinant of *end-of-sliding friction* at both 2 & 7 N (Fig. 8F).

## Discussion

The precise role of obesity in OA etiology remains unclear—due in part to insufficient studies of the direct influence of obesity-associated biomechanical factors on cartilage function. The present work leveraged the presence of tribological rehydration in the cSCA configuration [25] to examine the purely physical effects of elevated habitual (i.e., average) contact stresses (surrogates for body weight/obesity) on cartilage tribomechanics. The recent discovery of tribological rehydration demonstrated that articulation (i.e., sliding) can actively restore high levels of FLS and interstitial lubrication within articular cartilage by competing against load-induced fluid exudation [25, 34–36]. Thus, cSCA-based studies serve as a singular proxy for investigating the impact of contact stress on activity-mediated fluid exudation and recovery in cartilage.

Obesity causes elevated joint contact stresses [46–49], however, disagreements exist regarding appropriate healthy vs. obesity-mimicking contact stress inputs for ex vivo cartilage tribology studies. In normal weight individuals, peak cartilage contact pressures can exceed 5 MPa during gait [21], increasing with weight gain [39]. However, exposure of cartilage to these peak contact pressures is spatiotemporally brief—occurring for < 1/4th of the gait cycle [50] in a migrating contact manner [51, 52]. As a well-hydrated multi-phasic material [19], cartilage appears tribomechanically optimized to resist such rapidly applied/moving peak pressures [53]. In contrast, spatiotemporally averaged contact stresses, while only on the order of tenths of MPas during gait (and static loading) [21, 54, 55], unequivocally cause functionally detrimental interstitial fluid losses and lubrication compromise [19, 56, 57]. Thus, average habitual contact stresses would appear a more likely influencer of cartilage’s overall tribomechanical response than peak stresses. As a corollary, obesity-associated increases in average contact stresses should drive direct and predictable compromise of cartilage’s tribomechanical functions. Nevertheless, how and at what average contact stress cartilage’s critical activity-mediated fluid recovery processes and tribomechanical function(s) are compromised are unknown.

Since tribological rehydration is conserved across model species, anatomically smaller ovine stifles were used to study the influence of elevated contact stresses (up to 0.8 MPa) on activity-mediated fluid recovery and lubricity [36]. In PBS-bathed cSCA explants, increasing contact stress, from ~ 0.1 to 0.8 MPa, led to increased (i) start-of-sliding compressions (during time-controlled tribology tests) and (ii) end-of-sliding compressions (all tests), as well as (iii) the compromise—and occasional abolishment—of sliding-mediated compression recovery. Irrespective of whether sliding was initiated from low/zero (Study 1) vs. intermediate FLS (Study 2), ovine cartilage converged upon indistinguishable, load- and speed-dependent, dynamic sliding equilibrium compressions (refer to Supp. Fig. S9). However, the way explants approached these equilibria—reflected in their compression accumulation/recovery profiles—differed substantially and were predicated on (i) the tissues’ FLS state at the start-of-sliding—informed by biphasic theory and static load duration [19, 53], and (ii) competition between pressure-induced fluid exudation—again informed by biphasic theory—and articulation-mediated tribological rehydration—dictated by articulation speed (refer to Supp. Fig. S8).

Consider the following. When sliding is initiated upon near-full tissue depressurization, initial compression recoveries/rehydration can be extremely robust as competitive load-induced exudative flows in the tissue are zero (0). With further sliding, tribological rehydration replenishes interstitial pressures restoring competitive exudative flows until pressure-driven exudation and sliding-mediated rehydration are counterbalanced, and a stable sliding equilibrium is established. However, by near-totally depleting FLS prior to sliding [30]—a condition unlikely to occur in vivo [58, 59]—cartilage would experience the largest total compression exposures. In partially depressurized contacts (Study 2), non-zero exudative flows are present to compete with sliding-induced rehydration. Depending on initial conditions, two different compression trajectories (exhibiting reduced compression exposure) can be observed. If articulation is initiated from less than the sliding equilibrium compression (e.g., for 8 N), continued, albeit slower, compression occurs until sliding equilibrium (and peak compression) is established. If articulation is initiated from greater than the sliding equilibrium compression (e.g., for 4N) compression recovery is observed, but to a reduced degree. Should sliding be initiated from near maximal FLS, say immediately upon load application [25], explants would still converge upon the tissue load-dependent equilibrium sliding compression (e.g., ~ 45-50um at 2 N and ~ 110-120um at 8 N; Supp Table 3), but through a trajectory that minimizes total compression exposure. Such considerations have important mechanobiological implications regarding the compression/strain environment cartilage experiences (*see below*).

Investigating compression recovery in response to contact stress in our 80 mm/s studies—a speed approximating interfacial articulation during gait [34, 60]—highlights the tribomechanical sensitivity of cartilage to modest increases in habitual/average contact pressure. For sliding studies initiated from both low (Study 1) and intermediate levels of FLS (Study 2), key changes in tribological rehydration/compression recovery occurred beyond ~ 0.5 MPa. In fully depressurized explants, substantial recoveries of total compression/strain (40–90%) were seen at contact stresses ≤ 0.2 MPa. Despite relative compression recoveries decreasing monotonically with contact stress, absolute compression recovery increased up to ~ 0.5 MPa (36–75 μm, Fig. 4A, B) (Fig. 4C). At pressures above ~ 0.5 MPa tribological rehydration was exceedingly compromised, and ultimately abolished—at ~ 1 MPa (Fig. 4B, D). When sliding was initiated from intermediate to high levels of FLS (Study 2)—presumably a better representation of the in vivo environment—net compression recovery halted at ~ 0.45 MPa (Fig. 7H). Together, these studies indicate that even in the face of near continuous articulation-driven rehydration phenomenon—a “best case scenario” for fluid recovery—modest increases in average cartilage contact stresses—on the order of tenths of a MPa—are likely to markedly alter/compromise activity-mediated tribological rehydration, thereby increasing habitual tissue compressions and the presumed mechanobiological effects of such compressions on cartilage health (*see below*).

By correlating contact stress-dependent compression recovery outcomes (Study 1) with biphasic explant material properties (Figs. 5, 6) permeable, less stiff tissues experienced increased overall tissue compressions and reduced compression recoveries. Moreover, the contact stresses at which (i) maximal tribological rehydration occurred and (ii) tribological rehydration perfectly balanced exudation were strongly correlated with tissue compressive modulus and permeability. Stiffer explants in our cohort—having modestly higher compressive modulus and low permeability (as a result of natural tissue variation [61])—not only better resisted tissue compression under equivalent contact stresses but supported enhanced sliding-mediated compression recovery. Because Pearson product moment correlation analysis evaluates covariance among continuous variables based on linear relationships, it represents, as compared to Spearman rank-order correlation analysis, a more conservative—yet still robust—means of assessing the strength and direction of monotonic relationships within our dataset. Nevertheless, analysis of our data by Spearman correlation analysis (not shown) resulted in qualitatively identical behaviors. Again, these findings have important implications regarding the potential to predict the mechanobiological response(s) of articular cartilage under conditions of healthy vs. compromised tissue (*see below*).

The present study also highlights several critical, contact stress-dependent and independent frictional behaviors. As interstitial lubrication theory predicts, static loading drives FLS loss [19],} with predictable impacts on start-of-sliding kinetic friction coefficients (*μ*_*k*_) [62]. Across fully depressurized, PBS-bathed contacts (Study 1) start-of-sliding *μ*_*k*_ increased with applied contact stress (Fig. 4E) suggesting the generation of relatively higher frictional forces due to increased applied stress. In contrast, partially depressurized contacts (Study 2) experienced little change in start-of-sliding *μ*_*k*_ with increasing contact stresses (Fig. 7I), evidence of the combined effect of FLS and loading duration on start-of-sliding *μ*_*k.*_. Subsequently, sliding, because of tribological rehydration, restored and/or maintained FLS levels in a speed-dependent manner. Regardless of whether 80 mm/s sliding was started from low-, intermediate-, or high levels of FLS, very-rapid lubrication recovery (very-low *μ*_*k*_ values evolved within ~ 1 minute of the start-of-sliding, Figs. 3B, 7C, 8B) and largely consistent end-of-sliding *μ*_*k*_ were observed across explants (*μ*_*k*_ ~ 0.05, Figs. 4F, 7J, 8F) irrespective of applied contact stress. These results suggest that cSCA contacts articulated at physiologically informed sliding speed (e.g., 80 mm/s) can recover/maintain lubricity in a manner that is largely insensitive to contact pressures. Across our three studies the only variable that consistently influenced equilibrium *μ*_*k*_ was sliding speed (a rough surrogate of activity levels; Study 3, Fig. 8F), in line with all prior cSCA studies [25, 34, 38]. Whether contact pressures greater than 0.8 MPa (which exceed our devices load limit) might compromise lubricity/lubrication recovery in rapidly articulated cSCA contacts is an area of future investigation.

The fact that we observe tissue compression/strain accumulation behaviors, and to an extent compression recovery (i.e., tribological rehydration), to be far more sensitive to average contact stress than equilibrium friction coefficients (i.e., lubricity) in the cSCA raises several functional and mechanobiological considerations. The ability of cartilage to support swift rescue and maintenance of relatively low equilibrium friction coefficients (*μ*_*k*_ ~ 0.05 in PBS) across a range of physiologically informed average/habitual contact pressures suggests that interfacial friction/shear might be an overstated regulator of cartilage/chondrocyte health in vivo—or more precisely, a testing configuration specific artifact [37, 63]. This interpretation is supported by work demonstrating reductions in chondrocyte death/apoptosis in SCA tests upon the modest suppression of interfacial shear by synovial fluid (*μ*_*k*_ reduced from ~ 0.24 to ~ 0.08, a reduction which we obtained using PBS alone in the present study) [63] and by work showing that synovial fluid presence in rapidly slid cSCA contacts fosters a remarkable synergy between tribological rehydration, interstitial lubrication recovery, and synovial fluid presence [38]. Such synergy supports both vanishingly low equilibrium friction coefficients (μ_k_ ~ 0.003, i.e., superlubricity) and excellent explant viability on the benchtop [37]. In contrast, static compression/strain causes matrix catabolism and chondrocyte apoptosis; dynamic compressions/strains are pro-anabolic/chondroprotective [64]. These data suggest that usage environments—both ex vivo and in vivo—that expose otherwise healthy articular cartilage to chronically elevated average contact pressures are more likely to engender elevated average/habitual tissue strains, and, presumably, increased strain-dependent cellular dysfunction/catabolism [64], than lubrication compromise. This would appear true even in the face of articulation-mediated tissue rehydration (i.e., tribological rehydration). For example, in our cSCA explants, loaded and slid under increasing contact pressures (from ~ 0.1 to ~ 0.8 MPa), equilibrium lubricity was effectively insensitive to contact stress, while peak and sliding equilibrium compressions/strains increased > 2-fold between the lowest and highest contact stresses tested (SFigs. 2,6 & STable 3).

While the present study did not investigate the impact of synovial fluid on contact stress-dependent tribological rehydration and frictional responses in the cSCA, there is no reason to suspect that synovial fluid’s synergistic lubricating capacity (supporting equilibrium *μ*_*k*_ ~ 0.003) [38] would be compromised at the present contact pressures. Indeed, in support of this, recently published cSCA work demonstrates that (i) neither tissue strain nor start-of-sliding friction coefficients are altered by synovial fluid presence [38] and (ii) no differences in frictional behaviors are observed between naïve and OA-like cartilage (modeled via enzymatic degradation) tested in PBS or synovial fluid, yet OA-like cartilage exhibited marked increased tissue strain under identical testing conditions [65]. Given the exceedingly robust and physiologically replicative tribological behaviors of articular cartilage across our cSCA studies [25, 33, 36], it appears that obesity and physical inactivity, by driving elevated articular cartilage compressions/strains, and the mechanobiological response to them [64]—as opposed to compromise of lubricity—likely constitute the evolutionary “mismatches” driving direct physical compromise of our joints and the increased risk and prevalence of osteoarthritis in modern society [1].

## Conclusion

The present study was the first to investigate the tribomechanical response of cartilage to biofidelic average contact stress, sliding speed, and fluid load support conditions using the cSCA cartilage testing configuration. The divergent functional compression/strain and friction sensitivities to contact stress observed support our central premise that elevated habitual strains in the intact joint, resulting from chronically elevated contact pressures (due to obesity increasing net exudation) and/or increased static loading time (due to inactivity limiting rehydration behaviors), and not loss of lubricity, constitute a direct contributor to the functional and mechanobiological compromise of articular cartilage. In this manner, findings from cSCA-based approaches highlight the incredibly robust tribological nature of articular cartilage. They further suggest that a major—if not primary—consequence of elevated chronic contact pressures could be increased habitual cartilage strains. Should elevated habitual strain be accompanied by, or potentiate articular cartilage to chondro-dysfunction, as the literature suggests they do [64], a straightforward and testable driver of the dramatic increases in OA prevalence accompanying modern, postindustrial increases in obesity and inactivity/sedentariness might be identifiable [1, 2]. Furthermore, such a framework highlights the need to consider studies of human behavioral modification, namely weight loss/management and increased daily activity, as a means to reduce the evolutionary ‘mismatches’ driving the increased risk and elevated prevalence of OA in modern society.

## Supplementary Information

Below is the link to the electronic supplementary material.

Supplementary file1 (PDF 1227 kb)

## Data Availability

The data that support the findings of this study are available from the corresponding author, [CP], upon reasonable request.
