# Supplementary material for: Elevated Contact Stresses Compromise Activity-Mediated Cartilage Rehydration but not Lubrication
Source: Ann Biomed Eng. 2025 May 3;53(7):1672–88. doi: 10.1007/s10439-025-03708-z (PMC12185574; doi:10.1007/s10439-025-03708-z)
Supplement: Supplementary file 1 — Supplementary file1 (PDF 1227 kb) [file 10439_2025_3708_MOESM1_ESM.pdf]

Supplemental Materials

Supplemental Methods:

Characterization of Cartilage Biphasic Material Properties by Indentation: Micro-indentation was utilized to characterize the compressive modulus (E_{y-}), tensile modulus (E_{y+}), and unstrained permeability (k_0) of cSCA explant cartilage.

Indenter: A custom-built micro-indenter (**Supp Figure 1a**), having the same design principles as intenders previously designed and described by our team [1] was used to perform rate-controlled Hertzian-biphasic indentation tests under milli-Newton contact loads. The device comprised a custom calibrated cantilevered beam (1165 N/m spring constant) with an impermeable glass sphere (Φ=1.5mm) mounted at its tip. A nano positioning stage (Physik Instrumente [PI] LP, Q-545.240) with 26mm of travel and a resolution of 6nm was used to control indentation depth. Load-induced beam deflections were measured using a capacitance sensor (Lion Precision, CPL290, C3S; resolution 0.05-0.25nm). Indentation depth was defined as the difference between the stage displacement and the calibrated beam deflection. Applied contact force was calculated by multiplying the beam deflection by the beam's spring constant.

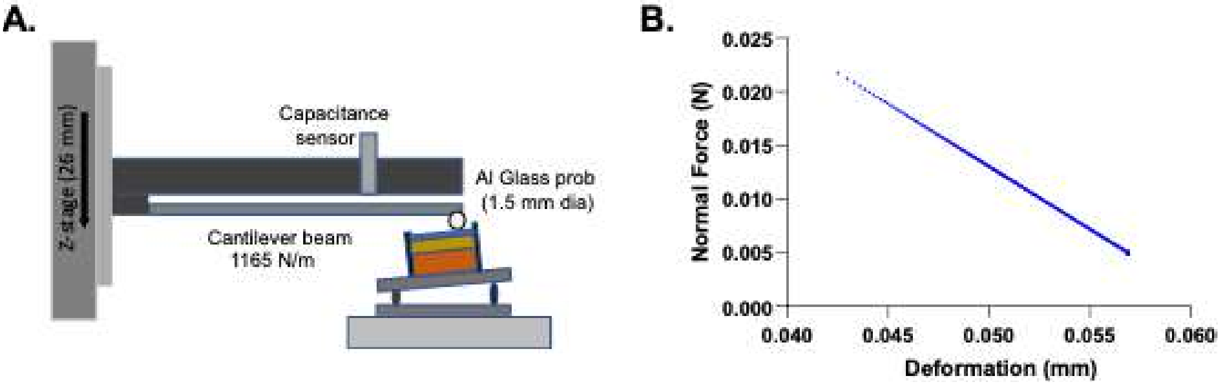

Supplemental Fig. 1: a) Schematic of the custom micro-indenter used to measure the biphasic material properties of cSCA explants. b) Representative normal load vs deformation traces extracted from an indentation test on an ovine cSCA explant used to determine the material properties of the cartilage via a hertzian biphasic model.

The indenter was controlled using LabView 7 (National Instruments) and stage position, beam deflection, indentation depth, and contact force were collected continuously at 20 Hz during the indentation, stress relaxation, and retraction/unloading testing phases for offline data analysis.

Indentation Tests: To perform an indentation test, the cSCA explant was affixed in a clamp atop a stage that allowed the indented surface to be positioned perpendicular to the axis of indentation. Once positioned, the cartilage surface was located by slowly driving the nano-positioning stage downward until the spherical probe contacted the cartilage and a 1-3mN load was registered. The stage was then retracted 5um to bring the probe out of contact with the cartilage surface. The explant was then indented at a rate of 50µm/s until a contact force of 20mN was reached; at this force, indentation depths varied from 60-90um and were always less than 20% of the sample thickness (**Supp Fig. 1B**). The probe was held at this indentation depth until the sample reached an equilibrium state (typically following 60-120 sec of stress relaxation) before being retracted at 50 um/s. Indentation was performed in the presence of PBS. Independent indentation

measurements were performed at three different locations (separated by a minimum of ~ 2mm) on each explant to account for potential spatial variation in tissue properties across each specimen.

Biphasic Tissue Property Calculation: Cartilage tissue material properties (E_{y-}, E_{y+}, & k_0) were obtained, as described previously,[2] by fitting the recorded force-displacement curves using Hertz biphasic tissue theory.[3] The cartilage was assumed to be an isotropic, homogeneous, linearly elastic material, exhibiting a zero (0) Poisson ratio [4],[5] and strain-dependent permeability [6]. Since the subchondral bone serves to effectively stiffen the articular cartilage the raw indentation data was corrected for substrate effects (i.e., tissue thickness) [7] using the method described by Moore and colleagues.[1],[4] The outcomes of the three independent indentation measurements performed on each explant were averaged to generate a single explant-specific set of material properties.

Akaike Information Criteria Analyses: The Akaike information criterion (AIC) is an information theory-based approach that allows identification, based upon statistical likelihood, the data model—from a collection of candidate models—that provides the highest ‘quality’ fit of experimental data.[8] When empirically derived data are represented using a statistical model (*i.e.*, an equation), the chosen model is unlikely to perfectly represent the underlying behavioral of the data, resulting in loss of information.[8] AIC allows one to estimate the relative quality of a candidate model by quantifying the degree of information lost when that model is applied; higher quality models are associated with less information loss. Mathematically, AIC assesses the degree of information loss by considering both the goodness-of-fit of the model and model simplicity, its formulation is as follows.

For each candidate model, an AIC value can be determined by:

$$AIC = 2k - 2\ ln\ ln\ (L)$$

Where k is the number of estimated parameters in the model, and L = the maximum likelihood function of the model.

For data sets with small sample size, a corrected AIC (AICc) approach should be used:

$$AICc = AIC + \frac{2k^2 + 2k}{n - k - 1}$$

Where n is the sample size.

Thus, given a set of **M** candidate models, the relative likelihood (P) that the nth model minimizes the predicted loss of information compared to the model with the minimum AIC value is:

$$P = e^{\frac{AIC_{min} - AIC_n}{2}}$$

Where AIC_{min} is the minimum AIC value observed among the candidate models, and AIC_n is the AIC value of the nth candidate model.

To identify the statistical data model(s) that provided the highest quality fit of the measured recovered compression vs. contact stress data within Study 1, AIC assessment was performed using GraphPad Prism (version 9.4.1, GraphPad Software). Five candidate equations were evaluated, these being:

1) A second-order polynomial constrained with a (0,0) root.

$$\delta_{rec} = ax + bx^2$$

2) An unconstrained second-order polynomial.

$$\delta_{rec} = c + ax + bx^2$$

3) A constrained straight line with a (0,0) intercept.

$$\delta_{rec} = ax$$

4) A hyperbola.

$$\delta_{rec} = \frac{\delta_{max} * x}{k + x}$$

5) A Gaussian distribution.

$$\delta_{rec} = \delta_{max} * e^{\left(-0.5*\left(\frac{x-\mu}{\sigma}\right)^2\right)}$$

These candidate models were selected based on their underlying mathematical behaviors conforming to both i) the known biphasic characteristics of the tissue (*e.g.*, zero compression at zero load/stress, and non-negative compressions at non-zero loads/stresses) and ii) the qualitative behavior of the recovered compression vs. contact stress data observed throughout our cSCA studies.

Supplemental Table 1: AIC-based Assessment of Candidate Model Fits Quality for Compression Recovery vs. Contact Stress Data (Study 1)

AIC assessment of the Likelihood of a Non-preferred Model Minimizing the Predicted Information Loss

Sample #	Constrained 2nd Order Polynomial			Un-Constrained 2nd Order Polynomial			Constrained 1st Order Polynomial			Hyperbola			Gaussian		
	R^2	AICc	*Likelihood*	R^2	AICc	*Likelihood*	R^2	AICc	*Likelihood*	R^2	AICc	*Likelihood*	R^2	AICc	*Likelihood*
1	0.77	35.17	10.6%	0.81	64.12	0.0%	0.4305	30.68	***Preferred***	0.79	34.72	13.3%	0.82	63.69	0.0%
2	0.96	29.37	***Preferred***	0.96	59.2	0.0%	0.0525	37.89	1.4%	0.90	34.46	7.8%	0.98	55.56	0.0%
3	0.89	32.26	1.1%	0.97	54.38	0.0%	0.8768	23.19	***Preferred***	0.89	32.43	1.0%	0.97	55.18	0.0%
4	0.97	23.87	3.6%	0.98	53.06	0.0%	0.9543	17.24	***Preferred***	0.97	23.65	4.1%	0.98	53.54	0.0%
5	*d.n.c.*	*n.a.*		0.63	54.71	0.0%	*d.n.c.*	*n.a.*		0.48	26.71	***Preferred***	0.59	55.29	0.0%
6	0.91	35.19	***Preferred***	0.96	60.83	0.0%	*d.n.c.*	*n.a.*		0.09	49.27	0.1%	0.92	64.72	0.0%
7	0.72	32.57	***Preferred***	0.80	60.51	0.0%	*d.n.c.*	*n.a.*		0.01	40.22	2.2%	0.79	60.93	0.0%
8	0.95	36.52	***Preferred***	0.98	61.99	0.0%	*d.n.c.*	*n.a.*		0.29	53.07	0.0%	0.82	74.86	0.0%
9	0.73	36.81	8.9%	0.92	59.54	0.0%	0.3599	31.98	***Preferred***	0.65	38.39	4.1%	0.91	59.92	0.0%
10	0.67	41.39	***Preferred***	0.68	71.19	0.0%	*d.n.c.*	*n.a.*		0.03	47.8	4.1%	0.60	72.48	0.0%
11	0.38	48.56	***Preferred***	0.49	77.41	0.0%	*d.n.c.*	*n.a.*		0.14	50.56	36.8%	0.62	75.58	0.0%
12	0.48	37.75	***Preferred***	0.67	65.03	0.0%	*d.n.c.*	*n.a.*		0.32	39.36	44.7%	0.62	65.79	0.0%
13	0.66	36.59	1.8%	0.67	66.57	0.0%	0.5319	28.6	***Preferred***	0.66	36.68	1.8%	0.69	66.14	0.0%
	7 preferred			0 preferred			5 preferred			1 preferred			0 preferred		

Based upon AIC analysis, the recovered compression vs. contact stress response of 7 of the 13 explants in Study 1 was preferentially modeled using a 2nd order polynomial having a fixed (0,0) root constraint. Qualitatively, all these explants exhibited a peak/maximal compression response at intermediate contact stresses followed by a clear reduction in compression response at higher stresses. The recovered compression vs. contact stress response of the remaining 6 explants appeared preferentially fit by either a 1st order polynomial (straight line) with a y-intercept = 0 (5 explants) or a hyperbolic curve (1 explant). The responses of these explants were similar in that their contact stress-dependent compression recovery had a largely linear appearance over the constrained/limited range of applied loads (*e.g.*, 8N) and contact stresses (<0.75MPa) that could be applied with our testing device.

Given that more than half of the explants tested exhibited robust inverted U-shaped 2nd order polynomial behaviors and limited to no likelihood of being described by a 1st order model, it is highly probable that the remaining explants would have exhibited 2nd order polynomial responses had we been able to apply larger applied loads/contact stresses. Based upon this AIC analysis and understanding of both biphasic cartilage mechanics and tribological rehydration, we chose to report 2nd order polynomial-based fits for all our compression recovery vs. contact stress (and applied load) data.

References:

[1] A. C. Moore, B. K. Zimmerman, X. Chen, X. L. Lu, and D. L. Burris, "Experimental characterization of biphasic materials using rate-controlled Hertzian indentation," *Tribology International*, 2015, doi: 10.1016/j.triboint.2015.02.001.

[2] M. E. Kupratis, A. E. Gure, J. M. Benson, K. F. Ortved, D. L. Burris, and C. Price, "Comparative tribology II–Measurable biphasic tissue properties have predictable impacts on cartilage rehydration and lubricity," *Acta Biomaterialia*, vol. 138, pp. 375–389, 2022, doi: https://doi.org/10.1016/j.actbio.2021.10.049.

[3] A. C. Moore, J. F. DeLucca, D. M. Elliott, and D. L. Burris, "Quantifying Cartilage Contact Modulus, Tension Modulus, and Permeability with Hertzian Biphasic Creep," *Journal of Tribology*, 2016, doi: 10.1115/1.4032917.

[4] A. C. Moore and D. L. Burris, "An analytical model to predict interstitial lubrication of cartilage in migrating contact areas," *Journal of Biomechanics*, vol. 47, no. 1, pp. 148–153, Jan. 2014, doi: 10.1016/j.jbiomech.2013.09.020.

[5] M. A. Soltz and G. A. Ateshian, "A Conewise Linear Elasticity mixture model for the analysis of tension-compression nonlinearity in articular cartilage.," *J Biomech Eng*, vol. 122, no. 6, pp. 576–586, Dec. 2000, doi: 10.1115/1.1324669.

[6] W. M. Lai, V. C. Mow, and V. Roth, "Effects of nonlinear strain-dependent permeability and rate of compression on the stress behavior of articular cartilage.," *J Biomech Eng*, vol. 103, no. 2, pp. 61–66, May 1981, doi: 10.1115/1.3138261.

[7] M. Stevanovic, M. M. Yovanovich, and J. R. Culham, "Modeling contact between rigid sphere and elastic layer bonded to rigid substrate," *IEEE Transactions on Components and Packaging Technologies*, vol. 24, no. 2, pp. 207–212, 2001, doi: 10.1109/6144.926384.

[8] J. E. Cavanaugh and A. A. Neath, "The Akaike information criterion: Background, derivation, properties, application, interpretation, and refinements," *Wiley Interdisciplinary Reviews: Computational Statistics*, vol. 11, no. 3, May 2019, doi: 10.1002/wics.1460.

Supplemental Data:

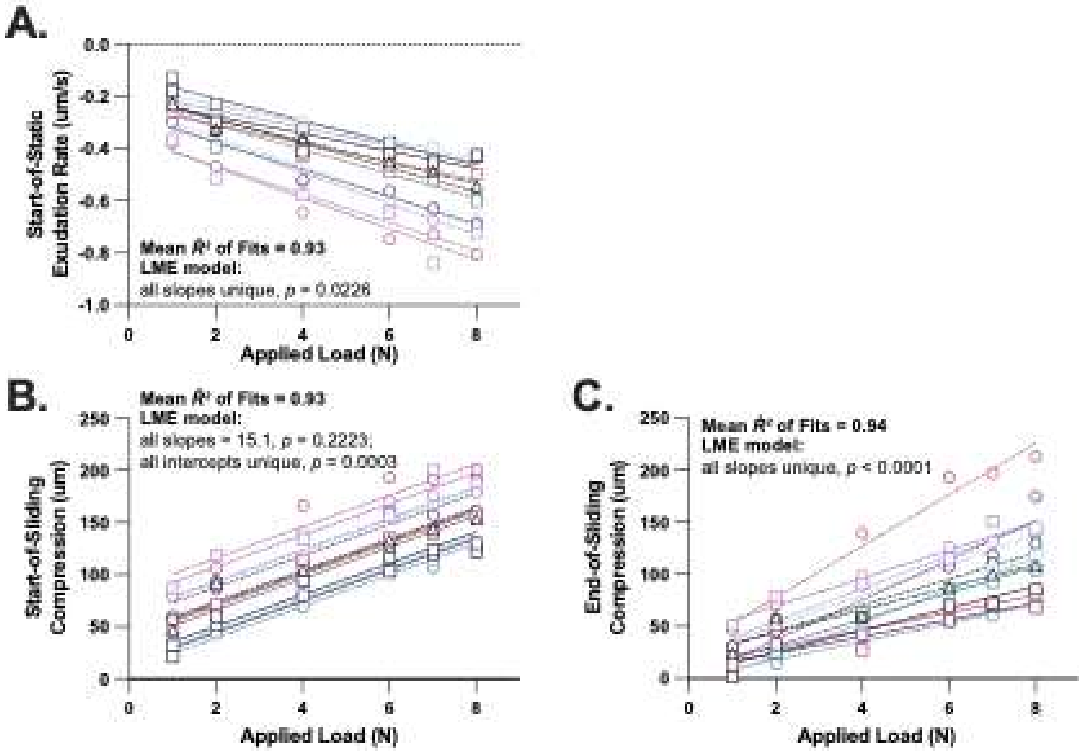

Supplemental Fig. 2*:* Load-dependent compression behaviors of individual cSCA explants upon loading, and during 80mm/s sliding initiated from quasi-static compression (Study 1, n = 13 explants). A) Fluid exudation rates of individual ovine cSCA explants upon the initiation of static loading (*a.k.a.*, *start-of-static*) increased linearly—becoming more negative—with increasing applied loads. **B)** *Start*- and **C)** *end-of-sliding compressions* increased linearly with increasing applied load when 80mm/s sliding was initiated from quasi-static compression. The labeling scheme for the cSCA explants depicted within these graphs is that same as shown in Figure 3C and 4A of the main Results section. The mean 1st-order (*i.e.*, straight line) goodness of fits ($\dot{R}^2$) averaged across all fits are indicted. LME analysis revealed explant-specific relationships among applied load and A) *start-of-static exudation rates*, and B) *start-* and C) *end-of-sliding compressions*. Explant-specific responses to unit increases in applied load were seen for *start-of-static exudation rate* and *end-of-sliding compression* magnitude responses (*i.e.*, different slopes, p_{slopes} = 0.0226 & <0.0001, respectively), while the *start-of-sliding compression* response to unit increases in applied load were indistinguishable among explants (p_{slopes} = 0.2223, slope = 15.1um/N); intercepts differed among specimens ($p_{intercepts}$ = 0.0003).

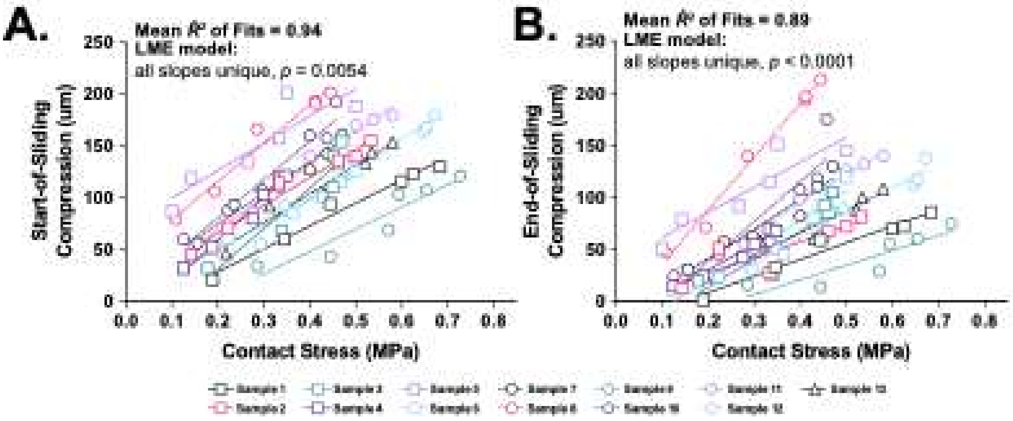

Supplemental Fig. 3: Contact stress-dependent compression behaviors of individual cSCA explants during 80mm/s sliding initiated from quasi-static compression (Study 1). A) *Start*- and **B)** *end-of-sliding compression* outcomes increased linearly in ovine cSCA explants in which sliding at 80mm/s was initiated from a condition of quasi-equilibrium compression. The labeling scheme for these graphs is that same as shown in Figure 3C and 4A. The mean 1st-order (*i.e.*, straight line) goodness of fits ($\dot{R}^2$) averaged across all fits are indicated. LME analysis revealed that the response of *start*- and *end-of-sliding compression* to unit increases in contact stress were explant-specific (p_{slopes} = 0.0054 & <0.0001).

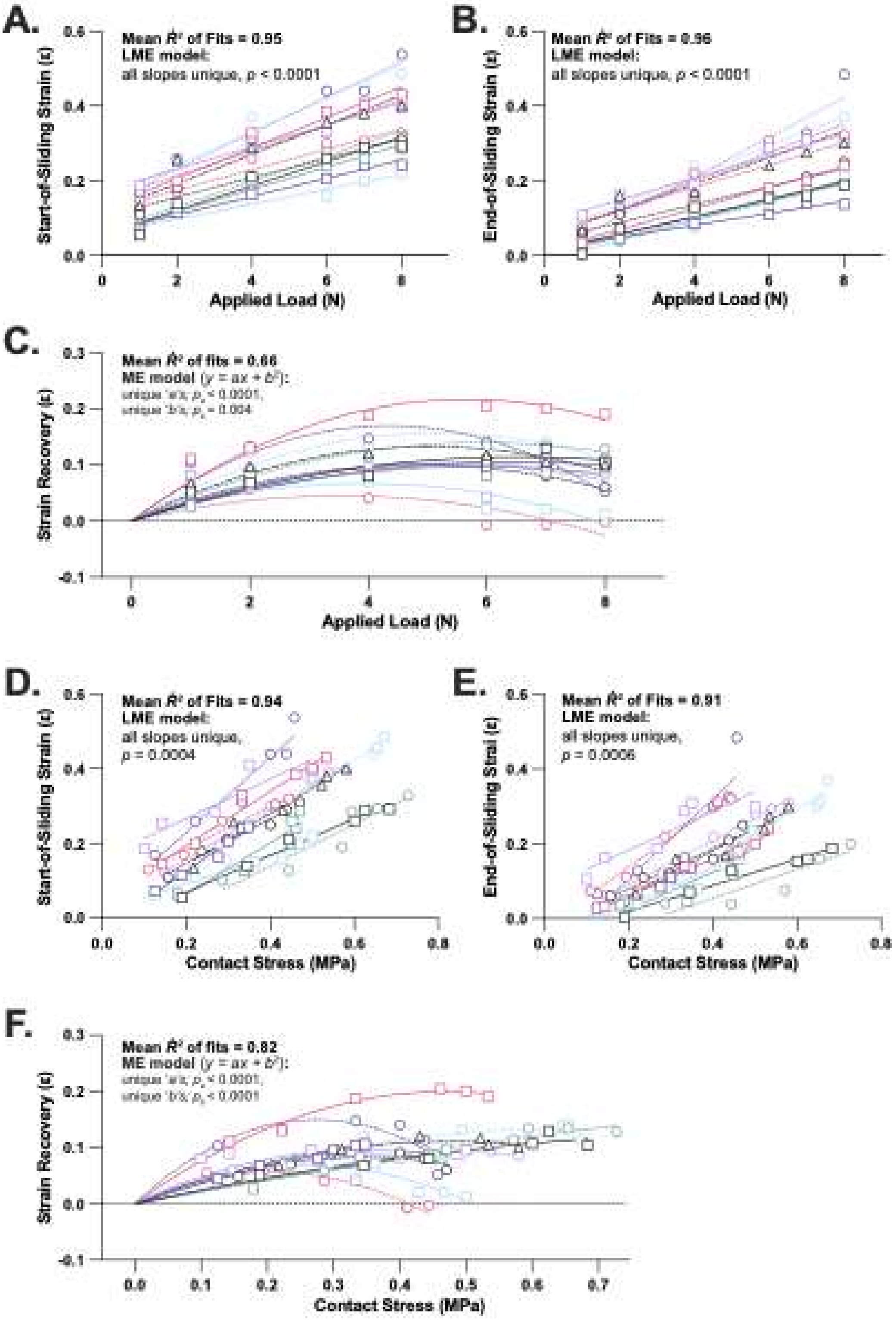

Supplemental Fig. 4: Applied load and contact stress-dependent strain recovery behaviors of cSCA explants slid at 80mm/s from quasi-static strain (Study 1). ME analysis demonstrated that **A)** *start-* and **B)** *end-of-sliding strains* within ovine cSCA explants increased linearly with applied loads when 80mm/s

sliding was initiated from a condition of quasi-static strain; and in a manner that was explant-specific (LME: p_{slopes} <0.0001). **C)** Sliding-mediated *strain recoveries* plotted as a function of applied load were best fit with a constrained 2nd-order polynomial (y = ax = bx^2) model and found to be explant specific (ME: p_a <0.0001, p_b = 0.004). **D)** *Start*- and **E)** *end-of-sliding strains* increased linearly in response to unit increases in contact stress, again in a manner dependent upon explant identity (LME: p_{slopes} = 0.0004 & 0.0006, respectively). **F)** Sliding-mediated *strain recoveries* plotted as a function of contact stress were similarly best fit with using a constrained 2nd-order polynomial model and found to be explant specific (ME: p_a <0.0001, p_b <0.0001). The labeling scheme for these graphs is that same as shown in Figure 3C and 4A. Mean goodness of fits ($\dot{R}^2$) averaged across all individual fits are indicted in each panel.

Supplemental Table 2: Material Properties of cSCA ovine explants assessed via indentation tests (Study 1)

Sample #	Compressive Modulus (MPa)	Tensile Modulus (MPa)	Permeability (mm^4/Ns)
1	0.56	3.24	0.0014
2	0.4	2.17	0.0031
3	0.565	2.53	0.0050016
4	0.285	1.52	0.004
5	0.156666667	1.5	0.009233333
6	0.214	1.34	0.0051
7	0.145	1.28	0.006
8	0.143	0.63	0.0126
9	0.49	1.75	0.003
10	0.126666667	1.1	0.0054
11	0.120333333	0.73	0.0095
12	0.4	1.45	0.0035
13	0.2	1.1	0.005675
Mean ± Std. Dev.	0.29 ± 0.17	1.56 ± 0.72	0.0057 ± 0.0031

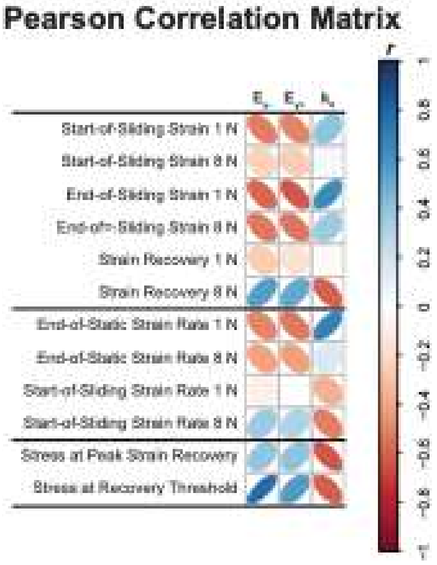

Supplemental Fig. 5: Relationships among cartilage biphasic tissue properties and strain outcomes for high-speed sliding initiated from quasi-static compression (Study 1). A) Pearson correlation matrix indicating the relationships observed between strain and strain recovery magnitudes and rates and measured biphasic tissue properties. For each pairwise comparison, both the box color and the oval ellipticity indicate Pearson correlation coefficient magnitudes.

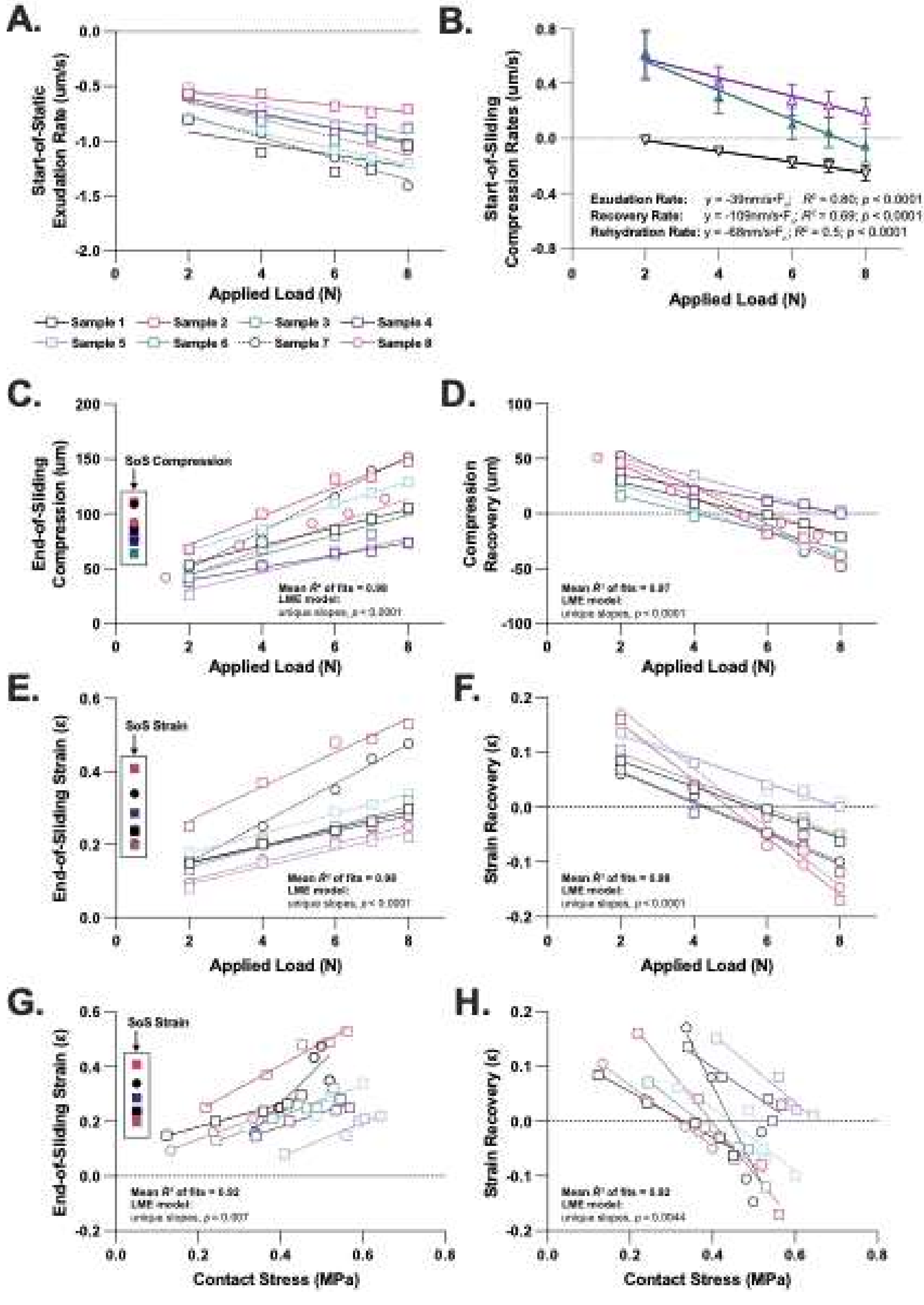

Supplemental Fig. 6: Additional load- and stress-dependent tribomechanical behaviors of ovine cSCA explants in response to 80m/s sliding initiated from intermediate levels of tissue compression/strain and FLS. A) *Start-of-static compression rates* of individual ovine cSCA explants

increased (became more negative)—as biphasic theory predicts—upon the application of larger loads. When sliding was initiated at a non-equilibrium target compression level (see panel Figure 7A and panel C), **B)** rates of fluid exudation at the start-of-sliding rose with increasing loads (slope = -39nm/s•N, p_{slope} <0.0001; mean data shown). Simultaneously, rates of compression recovery decreased (slope = -145nm/s•N, p_{slope} <0.0001). Negative *start-of-sliding recovery rates* appeared at loads ≥6N because rehydration rates, which also decreased with load (slope = -106nm/s•N, p_{slope} <0.0001), could not compete effectively against exudative forces at the start-of-sliding. Such behaviors led to *end-of-sliding compressions* **(C)** that increased and *compression recoveries* **(D)** that decreased with increasing loads. *End-of-sliding strains* **(E)** and *strain recoveries* **(F)** changed similarly in response to increasing loads. Target *start-of-sliding compressions/strains* upon which individual explant sliding was initiated are indicated in insets. LME analyses indicated that all load-dependent *end-of-sliding compression/strain* and *recovered compression/strain* relationships were specimen-specific (all p_{slopes} <0.0001). At applied loads ≤5.82±1.49N negative *compression recovery* occurred, meaning that net overall exudation occurred, leading to *end-of-sliding compressions/strains* that exceeded those at the start-of-sliding. Similarly, contact stress-dependent **G)** *end-of-sliding strains* and **H)** *strain recoveries* exhibited specimen-specific stress-dependent responses (LME: p_{slopes} = 0.007 & = 0.004, respectively). Based upon the strain recovery vs contact response, the mean contact stress at which net zero (0) strain recovery occurred (equivalent to *peak recovery stress* in Study 1) was 0.44±0.12MPa. Goodness of fit (R^2) or mean goodness of fits ($\dot{R}^2$) averaged across all individual fits are indicted in each panel.

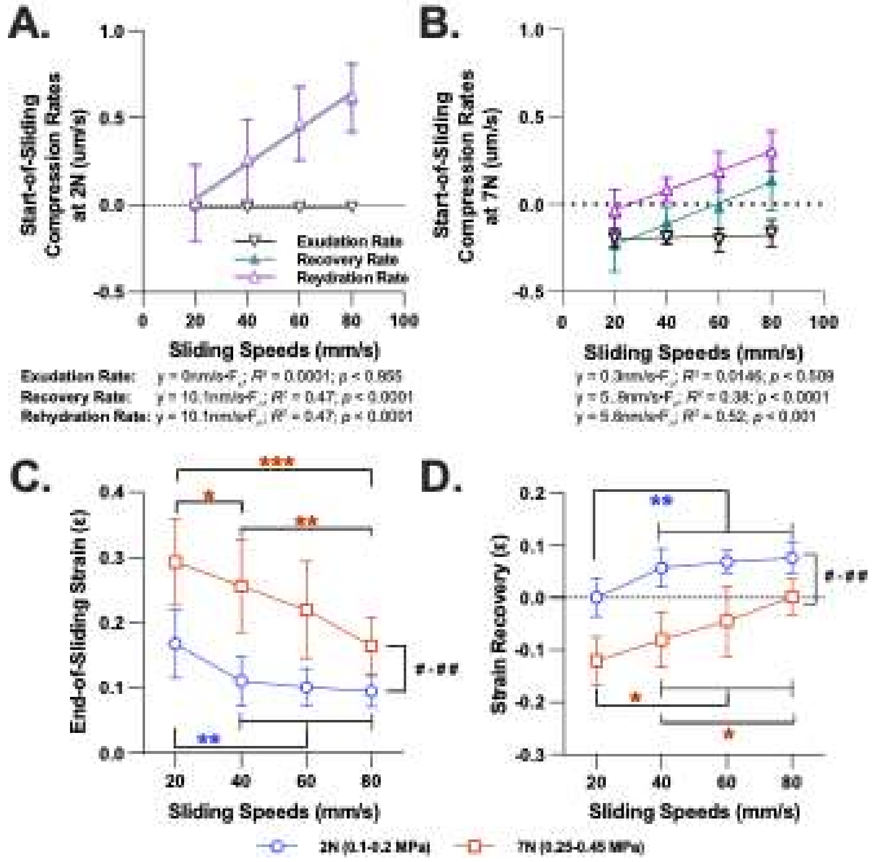

Supplemental Fig.7: Applied load- and sliding speed-dependent compression rates and strain behaviors of cSCA explants (Study 3). Assessment of 'linear' fluid exudation, recovery, and rehydration rates in ovine cSCA explants slid at various speeds under both **A)** 2N and **B)** 7N loads demonstrated the competitive nature of sliding speed-driven rehydration/recovery and load-induced exudation behaviors. As in Study 1, 20-minutes of loading at 2N resulted in the attainment of quasi-static compression conditions, thus, the resultant compression was taken to define each explant compression target magnitude. As such, *start-of-sliding exudation rates* at 2N were, expectedly, statistically indistinguishable from 0nm/s (p_{slope} = 0.955). Additionally, under the 2N load, *start-of-sliding recovery* and *rehydration rates* of approximately zero were observed for sliding at 20mm/s. However, as sliding speeds increased, *start-of-siding recovery* and *rehydration rates* increased (p_{slopes} <0.0001); with both having identical magnitudes due to conservation of volume (y = 10.1 nm/s for both rates). For tests conducted under 7 N loads, non-zero *start-of-sliding exudation rates* (~0.3 nm/s) were encountered because sliding was initiated at intermediate compression/FLS levels. Interestingly, while *rehydration rates* increased with increasing sliding speeds, these rates were markedly lower than those seen for the 2N load under identical sliding speeds. Data in panels A & B are shown as mean±95% CI, and the lines and statistical parameters associated with the best 1st order fits of the data are indicated. **C)** *End-of-sliding strains* decreased significantly with sliding speed and increased with applied load, whereas **D)** *strain recovery* increased with sliding speed and decreased with applied load. *Strain recovery* was negative for nearly all explants tested under 7N until a sliding speed of 80mm/s was achieved (under 7N only half of the explants exhibited zero or positive strain recovery at 80mm/s). In contrast, under 2N all explants demonstrated positive *strain recovery* (*i.e.*, tribological rehydration) at sliding speeds greater than 20mm/s. # - ## indicates statistically significant differences between 2 and 7N load for all speeds tests (p <0.05 to <0.01, respectively, two-way ANOVA). * - *** indicates statistically significant difference between noted speeds for a given applied load (asterisks color

indicates load level; $p < 0.05$ to < 0.001, respectively, two-way ANOVA). All groups ‘under’ the secondary brackets are significantly different from the indicated datum at the noted *p*-value.

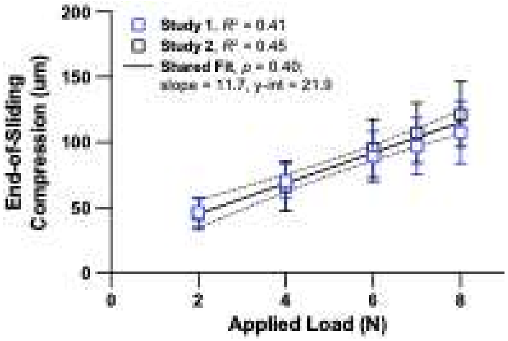

Supplemental Fig. 8: Dynamic sliding equilibrium behaviors of cSCA explants slid from quasi-static equilibrium vs. non-equilibrium compression levels (Study 1 vs. 2). Ovine cSCA explants slid at 80mm/s for 10-minutes exhibited common, load-dependent dynamic sliding equilibrium (*i.e.*, *end-of-sliding*) compression outcomes that were insensitive to sliding having been initiated from a state of quasi-static equilibrium (Study 1) or intermediate, non-equilibrium (Study 2) compression. Despite the large difference in *start-of-sliding compressions*, *end-of-sliding compression* outcomes were statistically indistinguishable among studies. Data shown as mean±95% CI. The common (shared) 1st-order best fit of both data sets is shown, along with the 95% CI of the best fit; p = 0.40, slope = 11.7um/N, intercept = 21.9um

Supplemental Table 3: Comparative start and end of sliding compression analysis for sliding initiation from quasi-static equilibrium compression (study 1), intermediate compression (study 2) and zero.

Supplementary Table 3

	cSCA Sliding Initiated from:					
	Quasi-static Equilibrium Compression (study 1)		Intermediate Compression (study 2)		Zero Compression	
**Load**	**SoS Comp.**	**EoS Comp.***	**SoS Comp.**	**EoS Comp.***	**SoS Comp.**	**EoS Comp.***
**2N**	~80um *(peak)*	~45um	~90um *(peak)*	~50um	0um	~45-50um *(peak)*
**8N**	~160um *(peak)*	~120um	~90um	~110um *(peak)*	0um	~110-120um *(peak)*

**In each case, the End-of-Sliding Compression reflected the sliding equilibrium compression state for the specified applied load (2 vs 8N) and sliding speed (80mm/s) .*

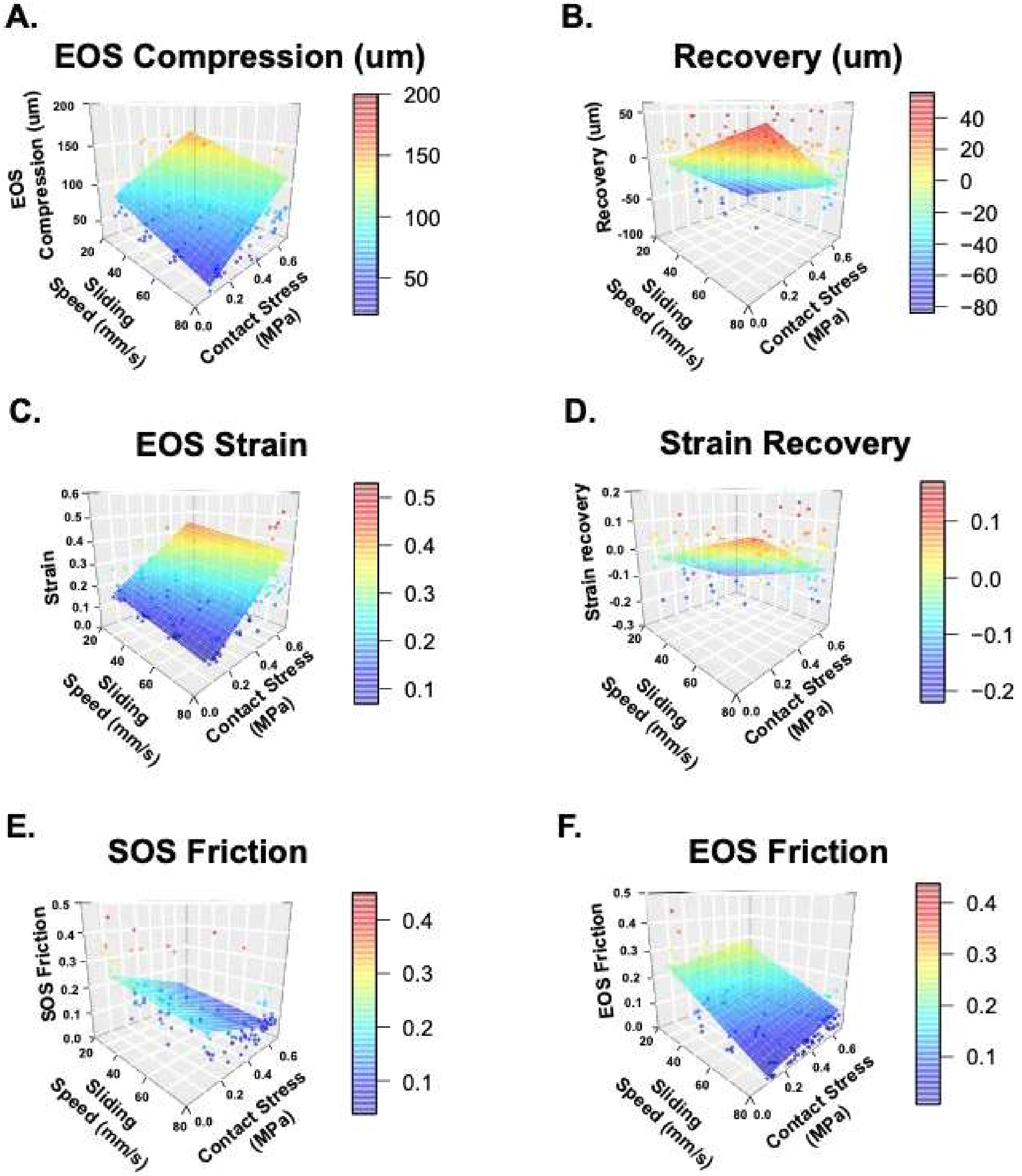

Supplemental Fig. 9: Three-dimension representation of the dependence of cSCA tribomechanical parameter outcomes as a function of sliding speed and contact stress. Data points represent the accumulation of experimental measurements from Study 2 and 3. Three-dimensional plots and the associated fits were generated using the Plot-3D package in R-studio. The color scales indicate the value of the associated tribomechanical parameter for ease of visualization. **A)** *End-of-sliding (EoS) compressions* increased with contact stress and decreased with sliding speeds, whereas **B)** *compression recovery* demonstrated the opposite behavior. **C)** *End-of-sliding strain* and **D)** *strain recovery behaviors* were consistent with those of see for *end-of-sliding compression* and *recovered compression*. In PBS-bathes cSCA contacts **E)** *start-of-sliding (SoS) frictions* tended to decrease with contact stress magnitude

but tended to remain constant across sliding speeds for a given stress level. **F)** *End-of-sliding frictions* were mainly controlled by sliding speeds and only modestly influenced by contact stress.
